# Subcutaneous application of *Helleborus niger* 12x in older patients with dementia—a retrospective cohort study

**DOI:** 10.3389/fpubh.2026.1758447

**Published:** 2026-03-25

**Authors:** Jan Vagedes, Angelika Hagen, Mohammad Oli Al Islam, Mohsen Sobh, Katrin Vagedes, Henrik Szőke, Matthias Kohl, Johannes Wilkens

**Affiliations:** 1 ARCIM Institute for Academic Research in Complementary and Integrative Medicine, Filderstadt, Germany; 2 Department of Neonatology, University Hospital Tübingen, Tübingen, Germany; 3 Geriatric Rehabilitation Center, Alexander von Humboldt Clinic, Bad Steben, Germany; 4 Department of Integrative Medicine, University of Pécs, Pécs, Hungary; 5 Institute of Precision Medicine, University Furtwangen, Villingen-Schwenningen, Germany

**Keywords:** cognitive impairment, traditional, complementary and integrative medicine, dementia, older adults patients, *Helleborus niger*, inpatients, subcutaneous application

## Abstract

**Introduction:**

Dementia is a neurodegenerative disease that affects more than 45 million patients worldwide. Treatment options for dementia include lecanemab for mild cognitive impairment, acetylcholinesterase inhibitors, which are mainly used for mild to moderate dementia, as well as N-methyl-D-aspartate receptor antagonists for patients with severe dementia or certain antipsychotics. Pharmaceutical approaches are limited by potential side effects. To alleviate symptoms or at least slightly improve cognitive functions, additional herbal medicines (HM) such as *Ginkgo biloba* and non-pharmacological approaches, e.g., behavioral, exercise, music, and reminiscence therapy, yoga, tai chi, or acupuncture, have been used with heterogeneous results. *Helleborus niger*, a plant containing multiple bioactive compounds such as ecdysteroids and bufadienolide, is used clinically for its immunomodulatory, anti-inflammatory, analgesic, and antioxidant properties. In a German hospital specializing in geriatric patients, an association between the subcutaneous application of *Helleborus niger* 12x and changes in dementia was observed.

**Methods:**

Retrospective study on the effect of subcutaneous application of *Helleborus niger* 12x for an average of 3 weeks in patients with dementia. Eligible patients were aged ≥ 60 years and treated at the Humboldt Clinic in Bad Steben, Bavaria, Germany, with two different treatment regimens: standard care with conventional medication alone (control group, CG) vs. standard care with conventional medication plus *Helleborus niger* 12x preparations (Helleborus group, HG). The primary outcome was the between-group difference in pre-post (T0 vs. T1) changes in the Mini-Mental State Examination (MMSE). Secondary outcomes were pre-post changes in the Dementia Detection test (DemTect), the Shulman clock-drawing test (CDT) and the Geriatric Depression Scale (GDS).

**Results:**

For the primary outcome, there was a statistically significant difference in MMSE between the groups (*p* < 0.001; *d* = 1.5) at T1 with significantly higher values in HG compared to CG. Regarding the secondary outcomes, significantly improved values were seen in HG compared to CG for DemTect, CDT and GDS with medium to high effect sizes.

**Conclusion:**

The study gives preliminary evidence that *Helleborus niger* 12x applied subcutaneously might improve cognitive function in patients with dementia at least during the three-week administration. Further randomized, blinded studies with longer follow-up are needed to confirm preliminary results.

**Clinical trial registration:**

Identifier, DRKS00033972.

## 1 Introduction

The prevalence of dementia is steadily increasing, not least against the backdrop of an aging population. The number of cases worldwide was estimated at 55 million in 2019 and could rise to 139 million by 2050 (1, 2). Alzheimer’s disease (AD) is the most common form of dementia and accounts for up to 70% of cases, followed by vascular dementia, Lewy body dementia, frontotemporal dementia and other forms (3). The symptoms of dementia usually begin with a gradual decline in cognitive abilities, often accompanied by changes in behavior and mood. The course of the condition is individual, but in most cases leads to an increasing dependence on support and care, often over many years, which in many cases is provided by family and friends and can place a considerable burden on these informal carers (4).

There is no known cure for dementia and the goals of the available treatment are to delay the progress of the irreversible neurodegeneration, to manage the behavioral changes and to improve the quality of life for the patient (5). The medications for dementia approved by the Food and Drug Administration (FDA) are acetylcholinesterase inhibitors (e.g., donepezil, galantamine and rivastigmine) and N-methyl-D-aspartate receptor antagonists (e.g., memantine) (6). Acetylcholinesterase inhibitors are effective in mild to moderate dementia, whereas memantine, an N-methyl-D-aspartate (NMDA) antagonist that targets the hyperfunction of the glutamatergic system, is approved for advanced dementia (7, 8). Also, some antipsychotics such as haloperidol, perphenazine, olanzapine, risperidone, aripiprazole and quetiapine have been shown to be partially effective in dementia (9). In recent years, lecanemab has been introduced for the treatment of early Alzheimer’s disease. Lecanemab was found to reduce markers of amyloid resulting in moderate beneficial effect on cognition and function at 18 months, but was associated with adverse events (10).

Two published systematic reviews and meta-analyses have shown that acetylcholinesterase inhibitors and memantine are slightly effective and beneficial in patients with dementia (11, 12). The meta-analysis by Jin et al. (11) included 146 randomized controlled trials involving 44,873 patients and concluded that memantine, donepezil and galantamine have moderate efficacy in behavioral and psychological symptoms of dementia, while rivastigmine is less effective. Moreover, acetylcholinesterase inhibitors (donepezil, galantamine and rivastigmine) are associated with a higher risk of adverse events, whereas memantine is considered safer (11).

The therapeutic spectrum in dementia treatment is further supplemented by behavioral therapy techniques such as exercise, music and reminiscence therapy, rehabilitation, cognitive intervention, yoga, tai chi, acupuncture, or acupressure, with heterogeneous results (9, 13).

Traditional medical systems such as Ayurveda and Traditional Chinese Medicine (TCM) have a long history of using herbal preparations to treat dementia. Plants such as ashwagandha (*Withania somnifera*), *Ginkgo biloba*, *Panax ginseng*, saffron (*Crocus sativus*), and turmeric (*Curcuma longa*) have demonstrated neuroprotective effects through antioxidant, anti-inflammatory, anti-amyloidogenic properties, neurotransmitter modulation, and various other neuropharmacological pathways, many of which are not yet fully understood (14, 15).

*Helleborus niger* L., Christmas rose, is a perennial plant from the Ranunculaceae family that grows on calcareous soils, in forests and thickets in the southern and eastern Alps of Europe (16). Some clinics and hospitals in Germany that specialize in integrative medicine use preparations made from *H. niger* in addition to conventional medications in oncology and neurology (17). Several compounds have been identified in the extract of *H. niger,* including bufadienolide, bufadienolide glycosides, 16-hydroxytaxisterone, taxisterone, 20-hydroxyecdysone, stachysterone B and shidasterone (18). These compounds have shown cytotoxic activity against leukemia cell lines and lung adenocarcinoma cell lines, while bufadienolide glycosides and ecdysteroid exhibit apoptotic properties through nuclear chromatin condensation, accumulation of sub-G1 cells, and activation of caspase-3/7 (18).

In TCIM (Traditional, Complementary, Integrative Medicine) literature, *H. niger* is used as a remedy for neurological and psychiatric symptoms such as stupor, mental sluggishness, confusion, post-traumatic or post-inflammatory brain disorders, and deep depression (19–21). According to Boericke, *H. niger* is a remedy for “sensory depression”; those affected are slow, have difficulty thinking, respond with delay, and are mentally absent (22). Scientific evidence is scarce overall. However, a randomized pilot study on the treatment of persistent mild traumatic brain injury showed significant improvements on some scales after treatment with *H. niger* (23).

At the Alexander von Humboldt Clinic in Bad Steben (Bavaria, Germany), *H. niger* in potentized form (12x) is frequently administered subcutaneously as an adjunct to standard treatment for patients with dementia, based on longstanding clinical experience. To the best of our knowledge, there are no studies that have investigated the effect of *H. niger* in patients diagnosed with dementia. We therefore retrospectively analyzed data from patients with dementia treated at the Alexander von Humboldt Clinic and compared two groups of patients, one with and one without *H. niger* 12x treatment. We hoped that this would provide data that could serve as a basis for further prospective clinical trials in the field of integrative dementia treatment.

## 2 Materials and methods

### 2.1 Study design

This study is a retrospective cohort monocenter pilot study. The study reporting follows the STrengthening the Reporting of OBservational studies in Epidemiology checklist (STROBE statement) (24).

### 2.2 Study setting and population

#### 2.2.1 Study setting

We analyzed data from 79 older adults with dementia who were treated at the Alexander von Humboldt Clinic between September 2020 and March 2022. The patients were admitted for geriatric rehabilitation following acute treatment in hospital and were assigned to one of the wards depending on bed availability. There was no special selection process. During their three-week inpatient stay, patients with dementia were treated in four different medical wards of the Humboldt Clinic. Two of these wards were headed by a senior physician who was open to phytotherapy/homeopathy, while the other two wards were headed by a senior physician who did not want to use phytotherapy/homeopathy. Depending on which of these wards the patients were admitted to, the senior physician prescribed subcutaneous injections of *H. niger* 12x (1 mL) two times a week in addition to the standard treatment program with conventional medications. *H. niger* plant extracts are produced according to the official protocol 34c (German Homeopathic Pharmacopeia, GHP) using finely crushed fresh plant material of the whole fresh flowering plants (rhizome, leaves and flowers), whey, and water in the ratio of 100:50:75 (w/w/w). 135 g water and 90 g whey are added to 180 g plant material per batch and further processed in accordance with protocol 34c to obtain an aqueous fermented extract. Based on a water content of the fresh plant material of 80%, this composition yields a concentration of 8.9 g/100 g (mass dry weight plant/mass extract) (25, 26). In accordance with the German Homeopathic Pharmacopeia, the mother tinctures are potentized to a dilution level of 12x, sterilized, and filled into ampoules without any additional additives.

For the analysis, the study population was divided into two groups according to the administration of *H. niger*: intervention group (Helleborus group, HG; receiving conventional medications plus *H. niger*) and control group (CG; receiving only conventional medications).

#### 2.2.2 Study population

Inclusion criteria

- Age ≥ 60 years
- Diagnosis of dementia according to the Mini-Mental State Examination (MMSE score ≤ 26)
- Impaired cognitive function measured with DemTect or clock-drawing test (DemTect score ≤ 12 or Shulman clock-drawing test score ≥3)

Exclusion criteria

- language barrier

### 2.3 Study procedure

#### 2.3.1 Data source

The Alexander von Humboldt Clinic uses the software Geridoc (GiB-DAT, Geriatrie in Bayern-Datenbank, Nürnberg, Germany) to store patient data. Clinical data was extracted from this database for the predetermined period (September 2020 to March 2022). Data extraction included patients’ demographic characteristics (e.g., age, gender, BMI, main diagnosis, secondary diagnoses, and medications prescribed at discharge) and the results of the cognition tests conducted at baseline (admission to the clinic, T0) and after 3 weeks (T1): Mini-Mental State Examination (MMSE), Dementia Detection test (DemTect), Shulman clock-drawing test and Geriatric Depression Scale (GDS-15). In addition, the medical data of the patients with dementia in both groups and the concentration and frequency of administration of *H. niger* preparations were extracted. The data extracted from the Geridoc database at the Humboldt Clinic was analyzed in anonymized form by the ARCIM Institute in Filderstadt, Germany.

#### 2.3.2 Outcome measures

Changes in patients’ cognitive status were examined using four outcome measures: MMSE, DemTect, clock-drawing test and GDS. All four measuring tools were routinely used at admission to the clinic (T0) and at discharge after 3 weeks (T1). The primary outcome was the between-group difference of the pre-post MMSE score changes (adjusted for the baseline values), while secondary outcomes were pre-post changes as assessed by the DemTect, the clock-drawing test, and the GDS.

### 2.4 Study instruments

#### 2.4.1 MMSE (mini-mental state examination)

The MMSE (27) is a validated tool widely used for diagnosing cognitive impairment in the older adults (28) and is an important parameter in assessing patient decline or the effects of memantine or acetylcholinesterase inhibitors on dementia (29–33). The MMSE includes items for assessing a patient’s spatial and temporal orientation, registration, attention and calculation, recall, and language (such as reading, writing, and copying of a design). The maximum possible score is 30, with lower scores indicating poorer cognitive state (34). The mean score for healthy people over the age of 65 is 27 (SD: 1.7); patients with AD lose three to four points for each year of their illness (35). The MMSE scores provide indicative information on the severity of dementia: 20 to 26 points—mild; 10 to 19 points—moderate; less than 10 points—severe (36). The examination takes about 10 min to complete (35). With a Cronbach’s alpha of 0.78, the MMSE shows good internal consistency (37).

#### 2.4.2 DemTect (dementia detection test)

The DemTect is a time-efficient validated screening tool that supplements the diagnosis of mild cognitive impairment, taking 8–10 min to complete (38). The test has a sensitivity of 80% and comprises five tasks: a word list, a number transcoding task, a word fluency task, digit span reverse, and delayed recall of the word list (39). The test scores achieved can be transformed according to age and education and are then independent of these factors (38). The maximum score is 18, with a score of 9 to 12 indicating mild cognitive impairment and a score below 9 suggesting dementia. Cognitive performance is considered age-adequate at a score of 13 to 18 (39). The DemTect shows good internal consistency with a Cronbach’s *α* of 0.79 (40).

#### 2.4.3 Shulman clock-drawing test

The validated clock-drawing test (CDT) is a basic neuro-psychometric instrument commonly used to assess cognitive impairment and its progression in patients with dementia. The test correlates with measures of disease severity and other dementia screening tests, such as the MMSE. In addition, it is relatively independent of culture, language, and education and has good interrater and test–retest reliability (41) as well as good internal consistency (Cronbach’s alpha coefficient: 0.90) (42). According to Shulman, patients are given a sheet of paper with a predrawn circle and are asked to write the numbers of a clock in the circle and set the time at 10 past 11. The Shulman scoring system is based on specified criteria regarding completeness and placement of the digits as well as correct time setting leading to a score of 1 for a perfect clock to 6 for a drawing that does not represent a reasonable reproduction of a clock (43).

#### 2.4.4 GDS-15 (15-item geriatric depression scale)

The Geriatric Depression Scale (GDS) is a validated self-report test to assess the non-somatic symptoms of depression, such as the psycho-social aspects and consequences. Somatic symptoms, such as sleep disorders, are a common feature of depression in younger people. However, they also frequently occur in non-depressed older individuals and therefore have less diagnostic value for depression in older age groups (44). Originally developed as a 30-item scale, the test was soon released as a 15-item short version, which facilitates use especially in cognitively impaired patients (45). All questions are answered simply with yes or no, and one point is given for a depressive response. Scores below five are considered normal, 5–9 suggest mild depression, and 10–15 indicate moderate to severe depression (46). A Cronbach’s *α* of 0.80 overall was reported for the internal consistency of the GDS-15 (47).

### 2.5 Statistical analysis

The demographic and clinical data of the included patients were extracted from the Humboldt Clinic’s clinic software Geridoc and anonymized on site. The statistical analysis of the anonymized data was then carried out by the ARCIM Institute, Filderstadt, Germany, in accordance with the relevant data protection regulations. Statistical analyses were conducted using the programming language R (version 4.5.1) (48) along with RStudio (Version 2025.05.1.513) (49). We present descriptive statistics for each group’s baseline values, including absolute and relative frequency for binary and categorical variables and means and SDs for continuous variables and counts of missing values. We analyzed our study’s primary outcome (MMSE at T1) using an ANCOVA, with the MMSE measurement at baseline (T0) included as a covariate. For the secondary outcomes, we analyzed DemTect, GDS-15, MMSE and CDT between two different time points and reported the results as mean±SD, mean difference (Δm), 95% confidence intervals (95CI) for continuous variable along with Cohen’s d effect size [d; R package: effsize (50)]. Pre-post analyses were also performed for three different degrees of dementia severity, with a classification into mild (MMSE = 20–26), moderate (10–19), and severe (below 10).

All statistical testing was two-tailed, with significance levels set at 5% and confidence intervals at 95%.

### 2.6 Ethics and data protection

The study was conducted in strict adherence to the principles set out in the Declaration of Helsinki. Data management was carried out in accordance with the Good Clinical Practice (GCP) guidelines and the European General Data Protection Regulation (EU-GDPR). The Ethics Committee of the Bavarian State Medical Association (Ethik-Kommission der Bayerischen Landesärztekammer) has confirmed that, due to the exclusive evaluation of anonymized data, there is no obligation to obtain approval in accordance with § 15 of the Professional Code of Conduct of the Bavarian Medical Association (letter no. 23102 dated March 12, 2024). The study was registered at the German Clinical Trials Register (Deutsches Register Klinischer Studien, DRKS-ID: DRKS00033972).

## 3 Results

### 3.1 Demographic data

Between September 2020 and March 2022, data was collected from a total of 79 patients, 54 (68%) of whom were in the Helleborus group (HG) and 25 (32%) in the control group (CG). 76 (96%) patients were female, there were only 3 male patients, all in the HG. The average age was 82.9 ± 7.2 years in the HG and 83.8 ± 5.5 years in the CG. The mean BMI was 26.9 ± 5.6 in the HG and 25.6 ± 5.0 in the CG (Table 1). All patients received physiotherapy regardless of their intervention group. Patients’ most frequently recorded primary diagnoses included diseases of the circulatory system (ICD-10 I00-I99; HG: 30.6%; CG: 21.3%), injury, poisoning and certain other consequences of external causes (ICD-10 S00-T98; HG: 19.9%; CG: 15%), and symptoms/abnormal clinical and laboratory findings (ICD-10 R00-R99; HG: 8.4%; CG: 7.5%). Further diagnostic examinations revealed additional health conditions. The most frequently recorded secondary diagnoses included diseases of the circulatory system (ICD-10 I00-I99; HG: 30.7%; CG: 32.3%), endocrine/nutritional/metabolic diseases (ICD-10 E00-E90; HG: 15.6%; CG: 17.7%) and diseases of the genitourinary system (ICD-10 N00-N99; HG: 7.2%; CG: 4.9%; Table 2).

**Table 1:** Demographic data and laboratory values of the study cohort.

	Total; n (%)/Mean ± SD	Helleborus group; n (%)/Mean ± SD	control group; n (%)/Mean ± SD	*p-*value
Demographic data				
Gender				0.570
Female	76 (96.2%)	51 (94.4%)	25 (100.0%)	
Male	3 (3.8%)	3 (5.6%)	-	
Age	83.1 ± 6.7	82.6 ± 7.2	83.8 ± 5.5	0.540
BMI^a^	26.5 ± 5.4	26.9 ± 5.6	25.6 ± 5.0	0.326
Laboratory values				
Leukocytes^b^	7.4 ± 2.5	7.4 ± 2.6	7.3 ± 2.3	0.943
Hemoglobin^c^	11.8 ± 1.9	11.8 ± 2.0	11.6 ± 1.7	0.651
Hematocrit	37.1 ± 5.5	37.3 ± 5.6	36.7 ± 5.2	0.632
MCV	96.4 ± 5.3	96.0 ± 5.4	97.2 ± 5.0	0.360
Thrombocytes	298.9 ± 113.9	294.5 ± 96.9	309.1 ± 148.6	0.667
Potassium	4.4 ± 0.5	4.4 ± 0.5	4.4 ± 0.5	0.881
Sodium	139.7 ± 3.8	139.8 ± 3.5	139.4 ± 4.5	0.700
Creatinine^d^	1.2 ± 1.2	1.3 ± 1.4	1.0 ± 0.4	0.196
CRP^e^	24.4 ± 24.9	24.3 ± 25.2	24.7 ± 24.7	0.948

Data are presented as either absolute and relative frequency, n(%) or Mean ± SD. Missing values are noted as follows: a: Helleborus group (HG) = 2 (3.7%), control group (CG) = 4 (16.0%); b: CG = 2 (8.0%); c: CG = 3 (12.0%); d: HG = 14 (25.9%); and e: HG = 1 (1.9%). BMI, Body Mass Index; MCV, Mean Corpuscular Volume; CRP, C-Reactive Protein. *p*-values were calculated using chi-square tests for categorical variables and t-tests for continuous variables.

**Table 2:** Diagnoses according to the international statistical classification of diseases.

Primary diagnoses	Total n (%) [*N* = 271]	HG n (%) [*N* = 191]	CG n (%) [*N* = 80]
Diseases of the circulatory system (I00-I99): e.g. *Hydropic decompensation with respiratory hypoxic insufficiency (I50.9), Bradycardic atrial fibrillation, emergency department presentation (no oral anticoagulation therapy so far) (I48.9), Coronary two-vessel disease with a history of percutaneous coronary intervention of the left anterior descending artery (1 drug-eluting stent) (I25.19) etc.*	75 (27.7%)	58 (30.6%)	17 (21.3%)
Injury, poisoning and certain other consequences of external causes (S00-T98): e.g. *Pertrochanteric femur fracture, right (S72.10), Recent fracture of the 11th thoracic vertebra (BWK11) (T14.20), Medial/basocervical neck of femur fracture, left (S72.00) etc.*	50 (18.5%)	38 (19.9%)	12 (15%)
Symptoms, signs and abnormal clinical and laboratory findings, not elsewhere classified (R00-R99): e.g. *Hypovolemic shock due to dehydration (R57.1), Recurrent falls (R26.8), Epistaxis (R04.0) etc.*	22 (8.1%)	16 (8.4%)	6 (7.5%)
Endocrine, nutritional and metabolic diseases (E00-E90): e.g. *Dehydration (E86), Severe hyponatremia and hypokalemia most likely due to diuretics (E87.1), Hyperglycemic uncontrolled diabetes mellitus type I under prednisolone therapy (E14.90) etc.*	16 (5.9%)	6 (3.1%)	10 (12.5%)
Diseases of the nervous system (G00-G99): e.g. *Parkinson’s disease (G20.90), Brachiofacial predominant left hemiparesis (G81.9), Cerebral atrophy with pronounced subcortical arteriosclerotic leukoencephalopathy (G31.9) etc.*	16 (5.9%)	12 (6.3%)	4 (5%)
Secondary diagnoses	Total n (%) [*N* = 594]	HG n (%) [*N* = 430]	CG n (%) [*N* = 164]
Diseases of the circulatory system (I00-I99): e.g. *Arterial hypertension with hypertensive heart disease (I10.90), History of atrial fibrillation with oral anticoagulation therapy (Marcumar) (I48.9), Status post-stroke with confusion, global aphasia, and retrograde amnesia (I64) etc.*	185 (31.1%)	132 (30.7%)	53 (32.3%)
Endocrine, nutritional and metabolic diseases (E00-E90): e.g. *Type 2 diabetes mellitus (E14.90), Folic acid deficiency (E53.8), Obesity (E66.99) etc.*	96 (16.2%)	67 (15.6%)	29 (17.7%)
Diseases of the genitourinary system (N00-N99): e.g. *Chronic kidney disease, Stage IV (N19), Urinary tract infection (N39.0), Prostatic hyperplasia (N40) etc.*	39 (6.6%)	31 (7.2%)	8 (4.9%)
Diseases of the musculoskeletal system and connective tissue (M00-M99): e.g. *Gonarthrosis (osteoarthritis of the knee) on the left (M17.9), Omarthrosis (osteoarthritis of the shoulder) (M19.91), Osteoporosis (M81.99) etc.*	37 (6.2%)	22 (5.1%)	15 (9.2%)
Diseases of the digestive system (K00-K93): e.g. *Antrum gastritis, axial hiatal hernia (K29.5), Constipation (K59.09), Severely pronounced diverticulosis in the sigmoid colon (K57.90) etc.*	36 (6.1%)	31 (7.2%)	5 (3.1%)

‘N’ represents the total number of diagnoses in each group. ‘HG’, Helleborus group (intervention); ‘CG’, control group.

During hospitalization, the most administered conventional medications (FDA-classified) were anticoagulants and thrombolytics (HG: 15.7%; CG: 22%), diuretics (HG: 16.5%; CG: 14.6%), and analgesics/antipyretics (HG: 13.9%; CG: 12.2%). Changes in medication were similar in both groups: newly introduced (HG: 31.3%; CG: 29.3%); dose increase/decrease (HG: 23.5%; CG: 19.5%); discontinuation of administration (HG: 35.7%; CG: 26.8%; Table 3A).

**Table 3A:** Most common medications in the study cohort during hospitalization.

Medications during hospitalization	Total n (%) [*N* = 156]	HG n (%) [*N* = 115]	CG n (%) [*N* = 41]
FDA-classified conventional medications			
Anticoagulants and thrombolytics, e.g., *Rivaroxaban (Xarelto), Enoxaparin (Clexane), Apixaban, etc.*	27 (17.3%)	18 (15.7%)	9 (22.0%)
Diuretics, e.g., *Torsemide (Torasemid), Furosemide, Hydrochlorothiazide (HCT), etc.*	25 (16.0%)	19 (16.5%)	6 (14.6%)
Analgesics, antipyretics, e.g., *Metamizole (Novalgin), Paracetamol (Acetaminophen), etc.*	21 (13.5%)	16 (13.9%)	5 (12.2%)
Opioid analgesics, e.g., *Tilidine, Oxycodone, Fentanyl, etc.*	11 (7.1%)	5 (4.4%)	6 (14.6%)
Antipsychotics, e.g., *Melperone, Pipamperon, Pipamperone, etc.*	6 (3.9%)	5 (4.4%)	1 (2.4%)
Antibiotics, e.g., *Trimethoprim/Sulfamethoxazole (Cotrim), Amoxicillin, Amoxicillin/Clavulanate (Augmentan), etc.*	5 (3.2%)	4 (3.5%)	1 (2.4%)
Antihypertensives, e.g., *Amlodipine, Valsartan, etc.*	5 (3.2%)	4 (3.5%)	1 (2.4%)
Hypoglycemics (oral/injectable), e.g.*, Insulin (long-acting), Sitagliptin (Januvia), etc.*	5 (3.2%)	5 (4.4%)	–
Antidepressants, e.g., *Citalopram, Duloxetine (Cymbalta), Opipramol, etc.*	4 (2.6%)	3 (2.6%)	1 (2.4%)
Sleeping drugs, e.g., *Zopiclone, etc.*	4 (2.6%)	2 (1.8%)	2 (4.9%)
Non-conventional or non-FDA listed medications			
Anthroposophic/herbal/homeopathic, e.g., *Ginkgo biloba (Ginkgo), Goldenrod (Solidagoren) drops, etc.*	2 (1.3%)	2 (1.7%)	–
Mineral/electrolyte supplements, e.g., *Potassium (Kalium), Sodium, Iron (II) sulfate (Ferro Sanol Duodenal), etc.*	16 (10.3%)	14 (12.2%)	2 (4.88%)
Other (non-FDA classification), e.g.*, Lanthanum Carbonate (Fosrenol), etc.*	1 (0.7%)	1 (0.9%)	–
Changes in medications during hospitalization	*N* = 156	*N* = 115	*N* = 41
*Newly introduced*	48 (30.8%)	36 (31.3%)	12 (29.3%)
*Changes in dosage (increase/decrease)*	35 (22.4%)	27 (23.5%)	8 (19.5%)
*Termination*	52 (33.3%)	41 (35.7%)	11 (26.8%)
*Other (*e.g.*, introduced and then discontinued)*	21 (13.5%)	11 (9.6%)	10 (24.4%)

‘N’ represents the total number of medications reported in each group. ‘HG’, Helleborus group (intervention); ‘CG’, control group.

In addition to the medications administered during hospitalization, the most prescribed conventional medications (FDA-classified) at discharge were antihypertensives (HG: 10%; CG: 13%), diuretics (HG: 8.8%; CG: 8.1%), and proton pump inhibitors (HG: 7.3%; CG: 6.9%; Table 3B).

**Table 3B:** Most common medications in the study cohort at discharge.

Medications at discharge	Total n (%) [*N* = 852]	HG n (%) [*N* = 591]	CG n (%) [*N* = 261]
FDA-classified conventional medications			
Antihypertensives, e.g.*, Ramipril, Amlodipine, Valsartan, etc.*	93 (10.9%)	59 (10.0%)	34 (13.0%)
Diuretics, e.g.*, Torasemide, Furosemide, Spironolactone, etc.*	73 (8.6%)	52 (8.8%)	21 (8.1%)
Proton pump inhibitors, e.g.*, Pantoprazole, Esomeprazole, etc.*	61 (7.2%)	43 (7.3%)	18 (6.9%)
Beta blockers, e.g.*, Metoprolol succinate, Bisoprolol, Carvedilol, etc.*	59 (6.9%)	40 (6.8%)	19 (7.3%)
Anticoagulants and thrombolytics, e.g.*, Apixaban, Clopidogrel, Rivaroxaban, etc.*	57 (6.7%)	41 (7.0%)	16 (6.1%)
Vitamins, e.g.*, Vitamin D, Folic Acid, Vitamin B Complex, etc.*	53 (6.2%)	38 (6.4%)	15 (5.8%)
Analgesics, e.g.*, Metamizol, Paracetamol, etc.*	51 (6.0%)	35 (5.9%)	16 (6.1%)
Antihyperlipidemics, e.g.*, Simvastatin, Atorvastatin, Fluvastatin, etc.*	37 (4.3%)	24 (4.1%)	13 (5.0%)
Hypoglycemics (oral/injectable), e.g.*, Insulin aspart, Metformin, Sitagliptin, etc.*	35 (4.1%)	26 (4.4%)	9 (3.5%)
Laxatives, e.g.*, Macrogol, Lactulose, etc.*	34 (4.0%)	21 (3.6%)	13 (5.0%)
Non-conventional or non-FDA listed medications			
Anthroposophic/herbal/homeopathic, e.g.*, Aconit Schmerzöl, Ginkgo biloba, Combination Preparation, etc.*	39 (4.6%)	35 (5.9%)	4 (1.5%)
Mineral/electrolyte supplements, e.g.*, Iron (II) glycine sulfate complex, Calcium Carbonate, etc.*	29 (3.4%)	15 (2.5%)	14 (5.4%)
Other (non-FDA classification), e.g.*, Alendronic Acid, Bimatoprost, Timolol, etc.*	16 (1.9%)	13 (2.2%)	3 (1.2%)

‘N’ represents the total number of medications reported in each group. ‘HG’, Helleborus group (intervention); ‘CG’, control group.

### 3.2 Statistical analysis of outcomes

#### 3.2.1 Primary outcome

At baseline (T0), there were no statistically significant differences in MMSE scores between the two groups (HG vs. CG). After adjusting for baseline MMSE scores (T0), there was a statistically significant difference in MMSE scores between the groups at T1 (F (1, 51) = 36.8, *p* < 0.0001), with the values in the HG (mean±SD: 22.2 ± 2.9; *n* = 54) being statistically significantly higher compared to the CG (mean±SD: 17.6 ± 3.0; *n* = 25) with a large effect size (Δm = 4.6; 95CI: 3.2, 6.1; *d* = 1.6).

For the within-group comparison, a statistically significant increase was observed in the HG (T0: 18.3 ± 3.9; T1: 22.2 ± 3.7; Δm = 3.9; 95CI: 3.0, 4.9; *d* = 1.0), whereas no significant change was observed in the CG (T0: 18.1 ± 3.0; T1: 17.5 ± 3.7; Δm = −0.6; 95CI: −2.0, 0.8; *d* = 0.2).

#### 3.2.2 Secondary outcomes

At T1, the DemTect score for the HG (9.7 ± 3.9) was significantly higher than that for the CG (5.7 ± 3.0), with a large effect size (Δm = 3.9; 95CI: 2.4,5.5; *d* = 1.1). No significant difference was observed at T0 between the two groups (HG: 6.6 ± 3.3; CG: 5.5 ± 3.1; Δm = 1.1; 95CI: −0.4, 2.6; *d* = 0.3). The between-group difference of the within-changes from T0 to T1 was statistically significant (Δm = 2.8; 95CI: 1.6,4.1) in favor of HG with a large effect size (*d* = 1.0).

For the CDT, there was a statistically significant difference between HG (2.9 ± 1.4) and CG (4.9 ± 1.3) at T1 with a large effect size (Δm = −2.0; 95CI: −2.7,−1.4; *d* = 1.4) as well as for the within changes from T0 to T1 with a large effect size (Δm = −1.9; 95CI: −2.4,**−**1.3; *d* = 1.4). No significant difference was found between the groups at T0 (HG: 4.5 ± 1.5; CG: 4.7 ± 1.7; Δm = −0.1; 95CI: −1.0, 0.7; *d* = 0.1).

Furthermore, at T1, there was a significant decrease in the GDS score between HG (2.4 ± 2.4) and CG (4.6 ± 3.5) with a large effect size (Δm = −2.2; 95CI: −3.8, −0.6; *d* = 0.8). At baseline, no significant differences were observed between HG and CG (HG: 5.2 ± 3.2; CG: 5.6 ± 3.4; Δm = −0.4; 95CI: −2.0, 1.3; *d* = 0.1). Within-changes from T0 to T1 differed statistically significantly between both groups in favor of HG with a medium effect size (Δm = −1.8; 95CI: −3.2, −0.5; *d* = 0.7).

**Figure 1:**
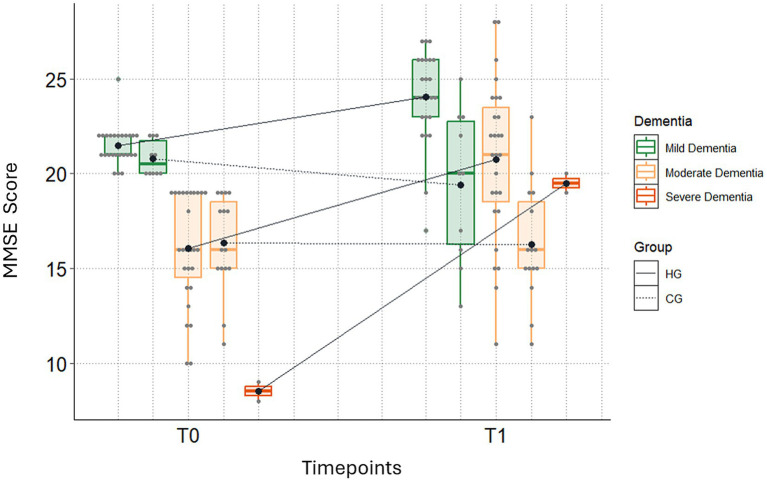
Changes in MMSE scores between T0 and T1, stratified by severity of dementia (there were no patients with severe dementia in the CG, but two in the HG). Please note: The severe dementia group is only represented by data points and a median line, as the available sample size was insufficient to create a complete box plot. All other groups are represented with standard box-and-whisker plots.

**Figure 2:**
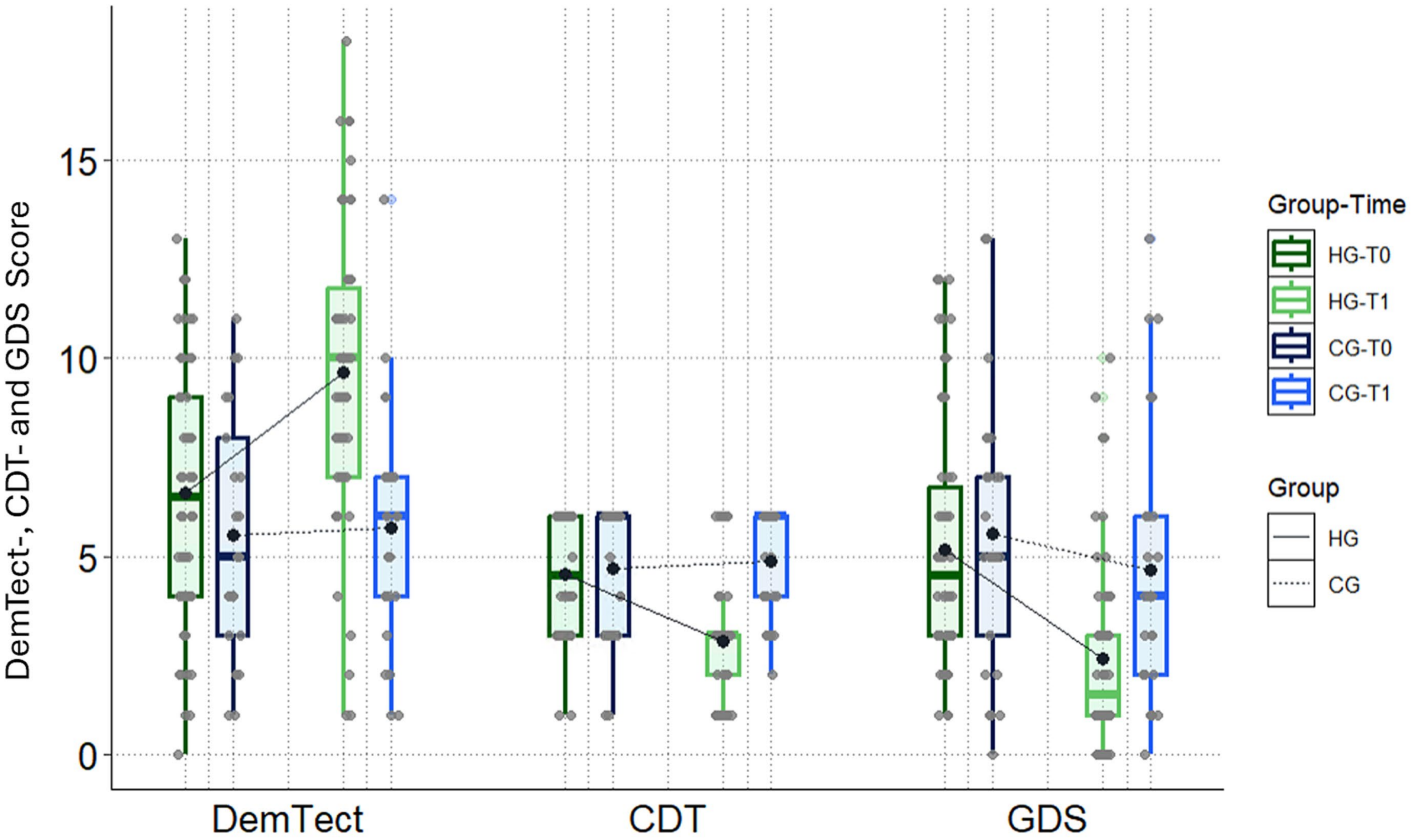
Secondary outcomes: changes in the dementia detection test (DemTect), the Shulman clock-drawing test (CDT), and the geriatric depression scale (GDS) for the Helleborus group (HG) and the control group (CG) between T0 and T1.

Regarding dementia severity classification, MMSE scores and secondary outcomes improved in the Helleborus group (HG) from T0 to T1 across mild, moderate, and severe stages of dementia (Table 4; Figures 1, 2). There were no patients with severe dementia in the CG, but two in the HG. The MMSE scores of these two patients improved from 9 to 20 and from 8 to 19, respectively. Additionally, both HG patients showed improvement in the secondary outcomes between T0 and T1. For one patient, the DemTect score improved from 4 to 7, the CDT score improved from 6 to 3, and the GDS score improved from 4 to 1. Similar values were seen for the other HG patient with severe dementia. The DemTect score improved from 2 to 8, the GDS score improved from 6 to 4, the CDT score remained unchanged with a value of 6 at T0 and at T1.

**Table 4:** Outcomes stratified according to the severity of dementia.

Mild dementia (MMSE 20–26)			
	Helleborus (*n* = 25)	Control (*n* = 10)	Helleborus vs. control
Mini-Mental State Examination (MMSE)			
T0	21.5 ± 1.0	20.8 ± 0.9	0.7(−0.1,1.4); d = 0.7
T1	24.0 ± 2.4	19.4 ± 4.0	**4.6(1.7,7.6); d = 1.6**
T1 vs. T0	**2.6(1.6,3.5); d = 1.4**	**−**1.4(−4.0,1.2); d = 0.5	**4.0(1.2,6.7); d = 1.4**
Dementia Detection Test (DemTect)			
T0	8.2 ± 2.7	6.6 ± 2.8	1.6(−0.6,3.8); d = 0.6
T1	11.3 ± 3.4	6.3 ± 1.6	**5.0(3.3,6.8); d = 1.6**
T1 vs. T0	**3.1(1.9,4.3); d = 1.0**	−0.3(−1.8,1.2); d = 0.1	**3.4(1.6,5.2); d = 1.3**
Shulman Clock-Drawing Test (CDT)			
T0	4.2 ± 1.5	4.0 ± 1.7	0.2(−1.2,1.5); d = 0.1
T1	2.6 ± 1.4	4.1 ± 1.5	**−1.5(−2.7,−0.4); d = 1.1**
T1 vs. T0	**−1.6(−2.2,−1.0); d = 1.1**	0.1(−0.3,0.5); d = 0.1	**−1.7(−2.4,−1.0); d = 1.3**
Geriatric Depression Scale (GDS-15)			
T0	5.0 ± 3.1	6.7 ± 3.9	−1.7(−4.7,1.3); d = 0.5
T1	2.7 ± 2.6	5.0 ± 4.1	−2.3(−5.3,0.7); d = 0.8
T1 vs. T0	**−2.3(−3.5,−1.0); d = 0.8**	−1.7(−3.5,0.1); d = 0.4	−0.6(−2.7,1.5); d = 0.2
Moderate dementia (MMSE 10–19)			
	Helleborus (*n* = 27)	Control (*n* = 15)	Helleborus vs. control
Mini-Mental State Examination (MMSE)			
T0	16.1 ± 2.9	16.33 ± 2.55	−0.3(−2.0,1.5); d = 0.1
T1	20.7 ± 4.2	16.27 ± 3.10	**4.5(2.2,6.8); d = 1.2**
T1 vs. T0	**4.7(3.2,6.2); d = 1.3**	−0.1(−1.8,1.6); d = 0.0	**4.7(2.5,6.9); d = 1.3**
Dementia Detection Test (DemTect)			
T0	5.4 ± 3.2	4.8 ± 3.1	0.6(−1.5,2.7); d = 0.2
T1	8.3 ± 3.8	5.3 ± 3.6	**2.9(0.5,5.3); d = 0.8**
T1 vs. T0	**2.9(1.6,4.1); d = 0.8**	0.5(−0.9,2.0); d = 0.2	**2.3(0.5,4.2); d = 0.8**
Shulman Clock-Drawing Test (CDT)			
T0	4.8 ± 1.5	5.1 ± 1.6	−0.4(−1.4,0.7); d = 0.2
T1	3.0 ± 1.4	5.4 ± 1.0	**−2.4(−3.1,−1.6); d = 1.9**
T1 vs. T0	**−1.7(−2.3,−1.2); d = 1.2**	0.3(−0.4,0.9); d = 0.2	**−2.0(−2.9,−1.2); d = 1.5**
Geriatric Depression Scale (GDS-15)			
T0	5.3 ± 3.4	4.8 ± 3.0	0.5(−1.5,2.6); d = 0.2
T1	2.2 ± 2.3	4.4 ± 3.3	**−2.3(−4.2,−0.3); d = 0.8**
T1 vs. T0	**−3.2(−4.1,−2.3); d = 1.1**	−0.4(−2.0,1.2); d = 0.1	**−2.8(−4.6,−1.0); d = 1.1**

Data are presented as either Mean ± SD or ∆Mean (95% confidence interval); effect size: Cohen’s d. Bold indicates 95% confidence interval does not contain zero. Comparisons between both groups are presented for mild and moderate dementia, as only two HG patients had a severe dementia with no severe dementia cases in the CG.

## 4 Discussion

The preliminary findings of this study are based on a retrospective evaluation of standardized questionnaire parameters collected at the time of clinic admission and discharge of patients with varying degrees of dementia. All patients were treated with standard therapy based on conventional medications. Some of the patients also received *H. niger* 12x as an additional therapy. The standardized, validated dementia questionnaires revealed no differences between the two groups in terms of dementia parameters at baseline (T0). However, the data indicated significant improvements in MMSE, DemTect, CDT, and GDS-15 scores in the HG at T1. No side effects or other adverse events were observed.

Although there is no known cure for dementia, a growing body of research has identified the efficacy of mind–body therapies, such as meditation, yoga, and Tai Chi, in reducing stress, enhancing neuroplasticity, and promoting overall well-being in patients with cognitive decline. These therapies have also been studied in conjunction with lifestyle modifications, including dietary changes and physical exercise (51, 52). Acupuncture has also been the subject of research regarding its potential to manage behavioral and psychological symptoms of dementia (BPSD). While the evidence is not conclusive, some studies suggest that acupuncture, when used in combination with psychotropic drugs, can improve symptom severity and reduce the need for pharmacological interventions (53, 54). Furthermore, studies have demonstrated that music therapy, including neurologic music therapy (NMT), can effectively reduce anxiety, depression, and agitation in dementia patients. Active engagement in music therapy sessions, particularly when facilitated by trained therapists, has also been demonstrated to enhance cognitive function and overall well-being (55, 56).

Among herbal remedies for treating cognitive decline and dementia, *Ginkgo biloba* has gained increasing attention and is a widely studied phytotherapeutic agent for dementia worldwide. Yang et al. conducted a systematic review and meta-analysis of RCTs evaluating Ginkgo alone or in combination with conventional medicines in mild cognitive impairment and AD (57). Although the results suggest a potentially beneficial effect of Ginkgo on cognitive function, daily activities, and global clinical assessment of patients, the evidence is inconclusive due to methodological weaknesses (57). An evaluation of 15 clinical trials revealed that 11 trials reported an improvement in cognitive performance and neuropsychiatric symptoms, while four trials found no benefit (58). In a pooled analysis, scores of the Short Cognitive Performance Test (SKT) improved by approximately 1.7 points in Alzheimer’s patients and 1.4 points in patients with vascular dementia, depending on the study. The Neuropsychiatric Inventory (NPI) improved by approximately 3 points, while no significant increase was observed in the global Mini-Mental State Examination (MMSE) (58). In a recent retrospective cohort study published by Yang, Koo and Kwak (59), the authors evaluated the effect of *Ginkgo biloba* alone in patients diagnosed with amyloid PET positivity, a prodromal stage of AD, over a 12-month period. Compared to the patients who received standard cognitive enhancers, the participants receiving oral Ginkgo monotherapy showed a significantly better outcome in cognition and daily functioning scores and a significant decrease in Multimer Detection System-Oligomeric Aβ levels, a biomarker reflecting Aβ oligomerization tendency (59).

Regarding *Crocus sativus* (saffron), a double-blind RCT involving 68 patients compared 30 mg of saffron per day over 12 months with 20 mg of memantine. At the end of the study, there was no significant difference in MMSE and Severe Cognitive Impairment Rating Scale (SCIRS) scores; the *C. sativus* arm improved by 1.88 points from baseline, while the memantine arm improved by 1.61 points (60), resulting in no clinically relevant advantage of *C. sativus* over standard therapy.

*Panax ginseng* was investigated in a randomized, placebo-controlled RCT involving older adults with mild cognitive impairment. Taking three grams of Korean red ginseng per day for 6 months showed no significant difference in MMSE and everyday functioning compared to placebo. Only certain memory tests (Rey Complex Figure Test) improved slightly (61). In open-label studies with Alzheimer’s patients, the combination of ginseng with conventional dementia medications showed improvements in MMSE and ADAS-Cog, but the studies were not placebo-controlled and had small sample sizes (61).

When using combination preparations such as SaiLuoTong (SLT)/WeiNaoKang (*Panax ginseng*, *Ginkgo biloba*, and *Crocus sativus*), the ADAS-Cog score decreased by 4.18 points in the SLT group compared to 1.18 points in the placebo arm (difference approx. 3 points) in a 16-week double-blind placebo-controlled study involving 325 patients with vascular dementia (62). A 52-week follow-up study showed a difference of 2.48–2.67 points in favor of SLT at week 26; after a group crossover, this advantage disappeared. A 12-week phase II SLT study in patients with mild cognitive impairment (MCI) showed significant improvements in the logical memory test and the trail-making test (executive function) with average differences of 1.37–1.56 points; however, a clinically relevant difference was not achieved (relevant threshold 3–5 points) (63). In summary, SLT appears to produce moderate improvements in cognitive tests, but the studies conducted to date are small and predominantly limited to Asian populations.

Traditional Chinese herbal formulas such as Danggui-Shaoyao-San (containing Danggui, *Angelica sinensis*, Baishao, *Paeonia lactiflora*, Fuling, *Poria cocos*, Baizhu, *Atractylodes macrocephala*, Zexie, *Alisma orientalis*, and Chuanxiong, *Ligusticum chuanxiong*) were examined in a meta-analysis that evaluated five RCTs on AD and four on vascular dementia. An average improvement of 4.60 points in the MMSE and 11.40 points in activities of daily living (ADL) was found for Danggui-Shaoyao-San compared to the controls (64). The results are of limited informative value due to the small number of cases, unclear randomization, and insufficient blinding.

Ashwaghanda (*Withania somnifera*) has a long history of use in Ayurvedic medicine to treat cognitive decline. In a randomized controlled pilot study evaluating the effect of ashwaghanda root extract in patients with mild cognitive impairment, Choudhary et al. observed significantly greater improvements in executive function, attention, and memory in the treatment group after 8 weeks (65). However, current data on the clinical efficacy of *W. somnifera* remain heterogeneous and incomplete, as noted in a systematic review by Ng et al. (66).

*Helleborus niger*, the Christmas rose, is characterized by the presence of ecdysteroids, among other constituents. Ecdysteroids, known for their role as arthropod steroid hormones, significantly influence acetylcholine activity by modulating acetylcholinesterase (AChE) activity. These compounds impact the nervous system by altering enzyme activity, which is critical for acetylcholine metabolism, affecting cognitive functions and developmental processes at least in insects. Ecdysteroids can induce AChE activity, as seen in Drosophila cell lines, where compounds such as *β*-ecdysone influence acetylcholine activity, suggesting a role in regulating cholinergic signaling (67, 68). On the other hand, some ecdysteroids, such as 20-Hydroxyecdysone-2,3,22-tri-O-acetate, act as inhibitors of AChE, demonstrating their capacity to modulate acetylcholine levels in different contexts (69). Additionally, in the midge *Chironomus tentans*, ecdysteroids promote morphological changes by increasing AChE activity, suggesting their involvement in morphogenesis through cholinergic signaling (70). Ecdysteroids, particularly ecdysterone, also exhibit neuroprotective and cognitive benefits, improving cognitive function and reducing oxidative stress through the Akt/GSK-3β/Nrf2 signaling pathway (shown in a mouse model) (71). The effect of ecdysteroids can be enhanced by combining them with high-intensity interval training (HIT), as shown in a rat model. Although ecdysterone separately improved memory impairments, spatial/passive avoidance learning, recovered hippocampal activity and prevented the hippocampal neuronal loss in rats, the combination with HIT led to a more effective amelioration in amyloid-beta-neuropathological changes (72).

While ecdysteroids are primarily recognized for their effects on insect development, their ability to modulate acetylcholine activity has broader implications for nervous system function and therapeutic use, emphasizing the need for further research to explore these applications in humans. In their 2021 overview regarding “Dietary Phytoecdysteroids,” Dinan et al. note that ecdysteroids appear to have the potential to target several possible sites in the early development of AD (73).

Although there are possible explanations at the phytotherapeutic level for the use of *H. niger* in neurodegenerative diseases, the proposed biological mechanisms are still the subject of research and need to be verified. Moreover, these mechanisms are not directly applicable to a 12x potentized preparation, as used in the present study. In the field of preclinical research (physico-chemical, *in vitro*, plant-based and animal-based test systems), a growing body of randomized and adequately controlled studies is emerging that substantiates the efficacy of potentized preparations (74–79). Furthermore, there is some evidence that medicinal plants in diluted and potentized form may be effective in a clinical setting, for example potentized *Arnica montana* in the context of postoperative recovery (80) or resin from *Larix decidua* Mill. (Pinaceae) for the treatment of ulcerating wounds (81). Regarding homeopathy meta-analyses, a meta-analysis published in 1998 concluded that individualized homeopathy might have a clinical effect over placebo (82). While some other meta-analyses either found too high a risk of bias in the homeopathy studies included or concluded that homeopathic medications have no efficacy beyond the placebo effect (83–85), a more recent systematic review of various meta-analyses concludes that the effect of potentized substances in a clinical context cannot be explained by the placebo effect alone (86).

*H. niger* in potentized form was used in a randomized controlled double-blind study by Chapman et al. (23), who treated 60 patients with persistent mild traumatic brain injury (MTBI) with either individually selected homeopathic remedies (including *H. niger*) or placebo. After 4 months, patients who received homeopathic remedies showed significant improvements on a Difficulty with Situations Scale (DSS; *p* = 0.009; 95% confidence interval = −0.895 to −0.15). A near-significant improvement was observed on the symptom assessment scale (*p* = 0.058) and in the 10 most common symptoms of MTBI (*p* = 0.027). The authors concluded that homeopathic treatment showed clinical benefit, but explicitly called for larger, independent studies (23).

Given this heterogeneous scientific background, it remains at least conceivable that *H. niger* 12x could have a clinical effect on neurodegenerative symptoms, as found in this retrospective analysis.

It remains unclear whether the findings presented can be explained by the ingredients of *H. niger,* as these were applied in diluted and potentized form (12x) and such substances continue to be the subject of controversy. There are alternative explanations that seem plausible and should be taken into account. In the context of inpatient geriatric rehabilitation, psychosocial factors are likely to have a significant impact. Patients strongly experience their dependency on the care and attention provided by physicians and nursing staff, a constellation that can be expected to generate considerable effects in terms of expectations, contextual and healthcare professional-patient interaction.

A well-known example in this context is the Pygmalion effect, originally described in 1968 by Rosenthal and Jacobson in the field of educational psychology (87). This phenomenon illustrates how one person’s expectations can influence the behavior and performance of another. In this landmark study, students perceived by their teachers as ‘high-potential’ performed better academically, even though this classification was completely random (87). Since then, the effect has been replicated in workplace (leader-subordinate relationships), health (caregiver-patient), and family contexts, demonstrating how the beliefs and expectations of others can unintentionally influence individuals, their decisions and performance (88–91).

Although the Pygmalion effect has traditionally been associated with human interactions, its experimental bases come from animal studies. Rosenthal and Fode conducted an earlier study in which students were told that some rats were ‘smart’ and others ‘dull,’ although all the animals were the same. The rats labeled as ‘smart’ learned more quickly and made fewer errors, demonstrating how experimenters’ beliefs impacted the animals’ outcomes (92).

Despite these findings, the Pygmalion effect has been the subject of controversy. Some critics have questioned the validity of Rosenthal’s original study, pointing to methodological flaws in IQ measurement and suggesting that the results could be explained by regression to the mean rather than by teachers´ expectations (93). Furthermore, subsequent meta-analyses have shown that the effect is often transient and limited, and frequently dissipates once teachers get to know their students better (94). Moreover, bidirectional interactions play a fundamental role in performance, particularly in social contexts (90). Since the patients in this study had been diagnosed with dementia, it remains open to what extent the positive effect of bidirectional interactions can be assumed and, therefore, to what extent the Pygmalion effect can be held responsible for the observed results.

Overall, it remains unclear whether our results are primarily due to the influence of expectations and interactions, the phytopharmaceutical effects of the ingredients of *H. niger*, the pharmaceutical dilution process, a combined effect of all three aspects, or simply a random effect. Furthermore, the effect sizes observed for our findings must be viewed critically and with caution, as they appear implausibly large for dementia outcomes over a three-week period and could be attributable to uncontrolled confounding. The high values could also be related to a regression to the mean effect (95, 96). For better understanding, further research should be conducted in this area to enable a profound, comprehensive understanding of a therapeutic approach that includes both pharmacological and psychosocial components as essential aspects of treatment.

This study has limitations that should be considered when interpreting its results. The study was designed as a retrospective cohort study, which inherently provides a lower level of evidence than randomized controlled trials (RCTs), as observational studies are prone to selection bias, information bias and confounding, among other things (97). The monocentric design makes it difficult to identify center-specific bias and confounding factors in the results. The lack of randomization leads to potential selection bias, as treatment assignment was ultimately based on the physician’s discretion rather than a random procedure, a limitation that is further exacerbated by the lack of blinding and placebo control. The decision to treat with *H. niger* 12x was based on clinical experience rather than a clear mechanistic rationale, which makes it difficult to evaluate the intervention in a scientific context. However, this is due to the fact that the data comes from a real-world clinical context and does not reflect the results of a prospective scientific study setting. Another limitation arises from the fact that the sample included only three male patients, all of whom were in the treatment group. Furthermore, the patient population was heterogeneous and included individuals with mild, moderate, and severe dementia, and no distinction was made between different subtypes of dementia, such as vascular dementia or AD. This variability in terms of severity and subtype of the disease complicates the interpretation of the results and limits their applicability to specific dementia subgroups. Overall, the monocentric design, small sample size, severe gender imbalance and heterogeneous severity of dementia limit the generalizability of the results. Finally, the short study duration of 3 weeks and the lack of follow-up assessment preclude the evaluation of possible long-term treatment effects and conclusions regarding safety or clinical relevance. In the absence of long-term observation, it therefore remains unclear whether any effects persisted over a longer period of time and whether there were any delayed responses or adverse effects that went unreported.

## 5 Conclusion

Our findings underscore the need for further prospective research, ideally in the form of non-randomized as well as randomized controlled trials with larger, more diverse samples, several physicians and longer follow-up periods, to provide more robust evidence of the potential effects and safety of *H. niger* in the treatment of dementia, and to elucidate possible effect-modulating factors. Especially the comprehensive cohort design (combination of a non-randomized and a randomized controlled trial in a single study) could be well suited to disentangle the different factors leading to the observed association between treatment with *H. niger* 12x and changes in dementia, which may be influenced by uncontrolled confounding and should be considered as hypothesis-generating observational evidence rather than therapeutic validation.

## Data Availability

The original contributions presented in the study are included in the article/supplementary material, further inquiries can be directed to the corresponding author.
